# Transgelin interacts with PARP1 in human colon cancer cells

**DOI:** 10.1186/s12935-020-01461-y

**Published:** 2020-08-03

**Authors:** Zhen-xian Lew, Hui-min Zhou, Yuan-yuan Fang, Zhen Ye, Wa Zhong, Xin-yi Yang, Zhong Yu, Dan-yu Chen, Si-min Luo, Li-fei Chen, Ying Lin

**Affiliations:** 1 Guangdong Provincial Key Laboratory of Malignant Tumor Epigenetics and Gene Regulation, Sun Yat-sen Memorial Hospital, Sun Yat-sen University, No. 107 West Yanjiang Road, Guangzhou, 510120 Guangdong China; 2 Department of Gastroenterology and Hepatology, Sun Yat-sen Memorial Hospital, Sun Yat-sen University, No. 107 West Yanjiang Road, Guangzhou, 510120 Guangdong China; 3 Department of Surgery, Guangzhou Concord Cancer Center, Guangzhou, 510045 China; 4 Department of Gastroenterology and Hepatology, The First Affiliated Hospital, School of Clinical Medicine of Guangdong Pharmaceutical University, Guangzhou, 510080 China; 5 Intensive Care Unit, Tongling People’s Hospital, Tongling City, 244000 Anhui province China; 6 Digestive Medicine Center, The Seventh Affiliated Hospital of Sun Yat-sen University, Shenzhen, 518107 China; 7 Department of Nephrology, Sun Yat-sen Memorial Hospital, Sun Yat-sen University, Guangzhou, 510120 China

**Keywords:** Transgelin, PARP1, Colon cancer, Rho signaling, Bioinformatics

## Abstract

**Background:**

Transgelin, an actin-binding protein, is associated with cytoskeleton remodeling. Findings from our previous studies demonstrated that transgelin was up-regulated in node-positive colorectal cancer (CRC) versus node-negative disease. Over-expression of *TAGLN* affected the expression of 256 downstream transcripts and increased the metastatic potential of colon cancer cells in vitro and in vivo. This study aims to explore the mechanisms through which transgelin participates in the metastasis of colon cancer cells.

**Methods:**

Immunofluorescence and immunoblotting analysis were used to determine the cellular localization of endogenous and exogenous transgelin in colon cancer cells. Co-immunoprecipitation and subsequently high-performance liquid chromatography/tandem mass spectrometry were performed to identify the proteins that were potentially interacting with transgelin. The 256 downstream transcripts regulated by transgelin were analyzed with bioinformatics methods to discriminate the specific key genes and signaling pathways. The Gene-Cloud of Biotechnology Information (GCBI) tools were used to predict the potential transcription factors (TFs) for the key genes. The predicted TFs corresponded to the proteins identified to interact with transgelin. The interaction between transgelin and the TFs was verified by co-immunoprecipitation and immunofluorescence.

**Results:**

Transgelin was found to localize in both the cytoplasm and nucleus of the colon cancer cells. Approximately 297 proteins were identified to interact with transgelin. The overexpression of *TAGLN* led to the differential expression of 184 downstream genes. Network topology analysis discriminated seven key genes, including *CALM1, MYO1F, NCKIPSD, PLK4, RAC1, WAS* and *WIPF1*, which are mostly involved in the Rho signaling pathway. Poly (ADP-ribose) polymerase-1 (PARP1) was predicted as the unique TF for the key genes and concurrently corresponded to the DNA-binding proteins potentially interacting with transgelin. The interaction between PARP1 and transgelin in human RKO colon cancer cells was further validated by immunoprecipitation and immunofluorescence assays.

**Conclusions:**

Our results suggest that transgelin binds to PARP1 and regulates the expression of downstream key genes, which are mainly involved in the Rho signaling pathway, and thus participates in the metastasis of colon cancer.

## Background

Colorectal cancer (CRC) is a frequent malignant tumor in the gastrointestinal tract worldwide. In 2018, it was ranked the third in terms of incidence (1.8 million new cases) and second in terms of mortality rate (881,000 deaths) [1]. Although mechanisms of CRC tumorigenesis and metastasis have been extensively studied, high mortality rates are still reported, especially in patients with advanced disease [1].

Tumor metastasis, the spread of cancer cells from the primary tumor to other areas of the body, is a complex process associated with remodeling of the cytoskeleton. The intracellular cytoskeleton requires a high degree of functional integration and coordination of actin (microfilament), microtubules and intermediate filaments [2–5]. However, abnormal expression of related genes or effector proteins may lead to the activation of various signaling pathways, thus promoting tumor metastasis [2–5].

Transgelin (also known as 22 kDa actin-binding protein, protein WS3-10 or smooth muscle protein 22 alpha) is an actin-binding protein with a molecular weight of 23 kDa and consists of 201 amino acids [6]. It is encoded by the *TAGLN* gene and composes of an N-terminal calmodulin homologous (CH) domain and a C-terminal calmodulin-like (CLIK) domain, which is closely related to actin binding activity [6]. Transgelin is broadly expressed in the vascular and visceral smooth muscle and is an early marker of smooth muscle differentiation [7]. Furthermore, transgelin is associated with the remodeling of the actin cytoskeleton and promotes the migration and invasion of cancer stem cells [8–10].

Recent studies have shown that besides the involvement in the regulation of actin nucleation, cellulose capping, fragmentation, actin monomer binding and other functions in the cytoplasm, actin-binding proteins are also involved in the formation of transcription complexes [11]. Transgelin has been shown to be a poor prognostic factor associated with advanced CRC [12] and it also promotes transforming growth factor β (TGF β)-dependent tumor growth and migration [13]. Moreover, results from in vitro experiments and a xenograft metastatic mouse model suggest that transgelin may be a promising therapeutic target for treating bladder cancer metastasis [14]. Therefore, we believe that transgelin may serve as a biomarker for tumor metastasis.

In our previous study, transgelin was up-regulated in the node-positive CRC versus node-negative disease [15]. While the up-regulation of transgelin promoted the metastasis of colon cancer cells, down-regulation substantially decreased the ability of cell invasion and metastasis [9, 10, 15]. In addition, gene expression profiling showed that over-expression of *TAGLN* affected the expression of the 256 downstream transcripts, which were closely related to cell morphology, migration and invasion [9]. We also found that transgelin localized in both the cytoplasm and nucleus of the cultured CRC cells and affected the expression levels of several epithelial to mesenchymal transition (EMT) associated genes [15]. Therefore, we hypothesized that transgelin may be a transcriptional regulator. However, the role of transgelin in colon cancer metastasis remains unknown.

Herein, we verified the nuclear localization of transgelin in different colon cancer cell lines. Approximately 297 proteins that are potentially interacting with transgelin were identified, of which 23 were DNA-binding proteins. Over-expression of *TAGLN* affected the expression levels of 184 genes. Seven key genes that mainly involved in the Rho signaling pathway were also identified. By analyzing the promoter regions of these key genes, poly (ADP-ribose) polymerase-1 (PARP1), a DNA-binding protein, was predicted to be the transcription factor (TF) of these genes. PARP1 was also among the 23 DNA-binding proteins that were perceived to interact with transgelin. The interaction between transgelin and PARP1 was further verified by immunoprecipitation and immunofluorescence.

## Materials and methods

### Cell lines

The human CRC cell lines, including RKO, SW480, HCT116, and LOVO were obtained from the Stem Cell Bank, Chinese Academy of Sciences (Shanghai, China). Cells were cultured in a minimum Eagle’s medium (MEM, Gibco, USA), Mccoy’s 5A medium (Gibco, USA) and Roswell Park Memorial Institute (RPMI) 1640 medium (Gibco, USA) with 10% fetal bovine serum (Gibco, USA). Cells were then cultured and incubated at 37 °C with 5% CO_2_.

### Immunofluorescence

Localization of endogenous transgelin in RKO, SW480, HCT116 and LOVO cell lines and the expression of PARP1 in RKO cells were determined by immunofluorescence. The primary antibody (anti-transgelin, 1:500, Abcam, USA; anti-PARP1, 1:500, Cell signaling technology, USA), secondary antibody (Alexa Flour 594 goat anti-rabbit IgG, Alexa Flour 488 goat anti-rabbit IgG, 1:500, Invitrogen, USA), and the VECTASHIELD mounting medium (Vector Laboratories, USA)) with 4′, 6-diamidino-2-phenylindole (DAPI) were used. The immunofluorescence images were taken and preserved under the laser scanning confocal microscope using a 63 × oil-immersion objective lens (Carl Zeiss, USA).

### Transfection

The SW480 and RKO cells were cultured in 12-well plates and transfected with pcDNA6/myc-His B-TAGLN-flag and pcDNA6/myc-His B-flag plasmids (Takara, Japan). The RKO cells were transfected with pENTER-TAGLN-Flag and pENTER-Flag control plasmids (Vigene Biosciences, USA) in the co-immunoprecipitation experiment. Transfection was conducted using Lipofectamine 2000/Lipofectamine 3000 (Thermo Fisher Scientific, USA). Cells were then harvested at 48 h after transfection for further analysis.

### RNA isolation, reverse transcription and polymerase chain reaction (RT-PCR)

Extraction of total RNA was performed using Trizol (Invitrogen) followed by reverse transcription (RT). Real-time polymerase chain reaction (PCR) was carried out using a Light Cycler 480 SYBR Green I Master mix (Roche, USA) on a Light Cycler 480 System (Roche, USA) according to the manufacturer’s instructions. The PCR conditions were as follows: 95 °C for 30 s, 35 cycles at 95 °C for 5 s, then 60 °C for 30 s. PCR primers are listed in Additional file 1: Table S2.

### Immunoblotting

The nuclear and plasma proteins from HCT116, SW480, LOVO and RKO cell lines were extracted using the NE-PER Nuclear and Cytoplasmic Extraction Reagents (Thermo Fisher Scientific, USA). The protein concentration of the extracted cytoplasmic and nuclear proteins was determined. Immunoblotting was performed with the primary antibody anti-transgelin (1:500, Abcam, USA, or 1:500, R&D, USA), anti-GADPH (1:400, Abcam, USA or 1:500, Cell signaling technology, USA), anti-PARP1 (1:500, Cell signaling technology, USA), anti-Lamin B1(1:1000, Cell signaling technology, USA), anti-flag (1:500, Cell signaling technology, USA) and the secondary antibody (horseradish peroxidase (HRP)-conjugated goat anti-rabbit or anti-mouse IgG, 1:30,000, Sigma-Aldrich, USA) or IgG Detector (IgG Detector Solution v2, HRP labeled, 1:1000, Takara, Japan). Antibody detection was performed using a chemiluminescence substrate and the protein bands were visualized with Syngene G: BOX Chemi XT4 fluorescence and chemiluminescence gel imaging system (Cambridge, UK).

### Immunoprecipitation

The RKO cells were cultured conventionally and transfected with pcDNA6/myc-His B-TAGLN-flag and pcDNA6/myc-His B-flag plasmids. The RKO cells were transfected with pENTER-TAGLN-Flag and pENTER-Flag control plasmids in the validation experiment. Cells were then harvested at 48 h after transfection for further analysis. Antibody immobilization, cell lysis, pretreatment of cell lysate with control agarose resin, immunoprecipitation, immunoprecipitation elution, and immunoblotting analysis were performed in sequence according to the protocol of the Pierce Crosslink Immunoprecipitation Kit (Thermo Fisher Scientific, USA). Anti-flag antibody (10ug, Sigma-Aldrich, USA, for the subsequent mass spectrometry; 1:50, Cell signaling technology, USA, for the validation experiment) and the control rabbit IgG (1:50, Cell signaling technology, USA) were used.

### Mass spectrometry

A fraction of the protein samples after immunoprecipitation was analyzed using SDS-PAGE and silver staining (Invitrogen, USA). Another fraction of the samples was used for high-performance liquid chromatography assay (EASY-nLC™, Thermo Fisher Scientific, USA) after filter-aided sample preparation (FASP) and enzymatic hydrolysis. The samples were then analyzed with a Q-Exactive Mass Spectrometer (Thermo Finnigan, USA). The mass/charge ratios of peptides and fragments of peptides were collected. Maxquant 1.3.0.5 software was used to retrieve the Uniprot database by using the raw file as source. The search in the database was set up with specific parameters (Enzyme, trypsin; De-Isotopic, True; Max Missed Cleavages, 2; Fixed modifications, Carbamidomethyl (C); Variable modifications, Oxidation (M); First search ppm, 20 ppm; Main search ppm, 6 ppm; Decoy database pattern, reverse; Min. Reporter PIF, 0.75; Peptides false discovery rate (FDR) ≤ 0.01; Protein FDR ≤ 0.01).

### Bioinformatics

#### Identification of differential expression genes (DEGs), functional enrichment and signaling pathway enrichment analysis

The relevant cDNA microarray data were obtained using the Affymetrix microarray technique based on our previous work [9]. Over-expression of *TAGLN* in RKO human colon cancer cells resulted in 256 downstream transcripts that were differentially expressed with at least a twofold change (P < 0.05). Among these, transcripts without gene symbols, gene database codes and duplicates were excluded. The remaining DEGs were screened for further bioinformatics analysis.

Using the Metascape tool (http://www.metascape.org/), the screening parameters were set as follows: P < 0.01 or 0.001 (Biological Process), participating genes ≥ 3 and enrichment factor > 1.5. We conducted functional and signaling pathway enrichment analysis of the DEGs referring to the gene ontology (GO), Kyoto Encyclopedia of Genes and Genomes (KEGG) pathway and Reactome databases.

#### Construction of the protein–protein interaction (PPI) network, topological analysis and key gene screening

The DEGs were simultaneously translated into proteins and the search tool for retrieval of interacting genes (STRING 10.0, https://string-db.org/) [16] was used for PPI analysis. Subsequently, relevant data was imported into the Cytoscape online software (http://www.cytoscape.org/) [17] and a PPI network was constructed. In this study, the degree centrality and intermediate centrality of the DEGs were calculated using the CytoHubba plug-ins. Those with values twofold higher than the overall average value were selected as the core genes in the network. In addition, the core modules were obtained with an MCODE plug-in (k-core = 2). The core genes and the genes included in the core modules were defined as the key genes. Key genes were further analyzed with Metascape for signaling pathway enrichment in KEGG and Reactome database using the same parameters previously mentioned.

#### Prediction of the TFs for the key genes

The TF evaluation model within the Gene-cloud of biotechnology information (GCBI) tools (https://www.gcbi.com.cn/) was used to predict the TFs for the key genes. Those with medium or high recommendations were selected and potential TFs were selected for further analysis. We then compared these potential TFs to the DNA-binding proteins identified in the mass spectrometry analysis.

#### Nuclear localization signal analysis

The sequences of the selected potential TF(s) were obtained from the Uniprot database (https://www.uniprot.org/) [18]. The classical nuclear localization signals (cNLS) Mapper (http://www.nls-mapper.iab.keio.ac.jp/) [19] was used to detect the nuclear localization signal of the potential TF(s).

### Statistical analysis

Statistical analysis was performed using the SPSS 20.0 software (IBM Corp., Armonk, USA). The relevant values were expressed as mean ± standard deviation (SD), and the significance of the difference between two groups was determined with the Student’s *t* test. Pearson correlation analysis was used to analyze the expression level of the key genes obtained from cDNA microarray and RT-PCR. P < 0.05 (bilateral) was considered statistically significant.

## Results

### Localization of transgelin in human colon cancer cell lines

**Fig. 1:**
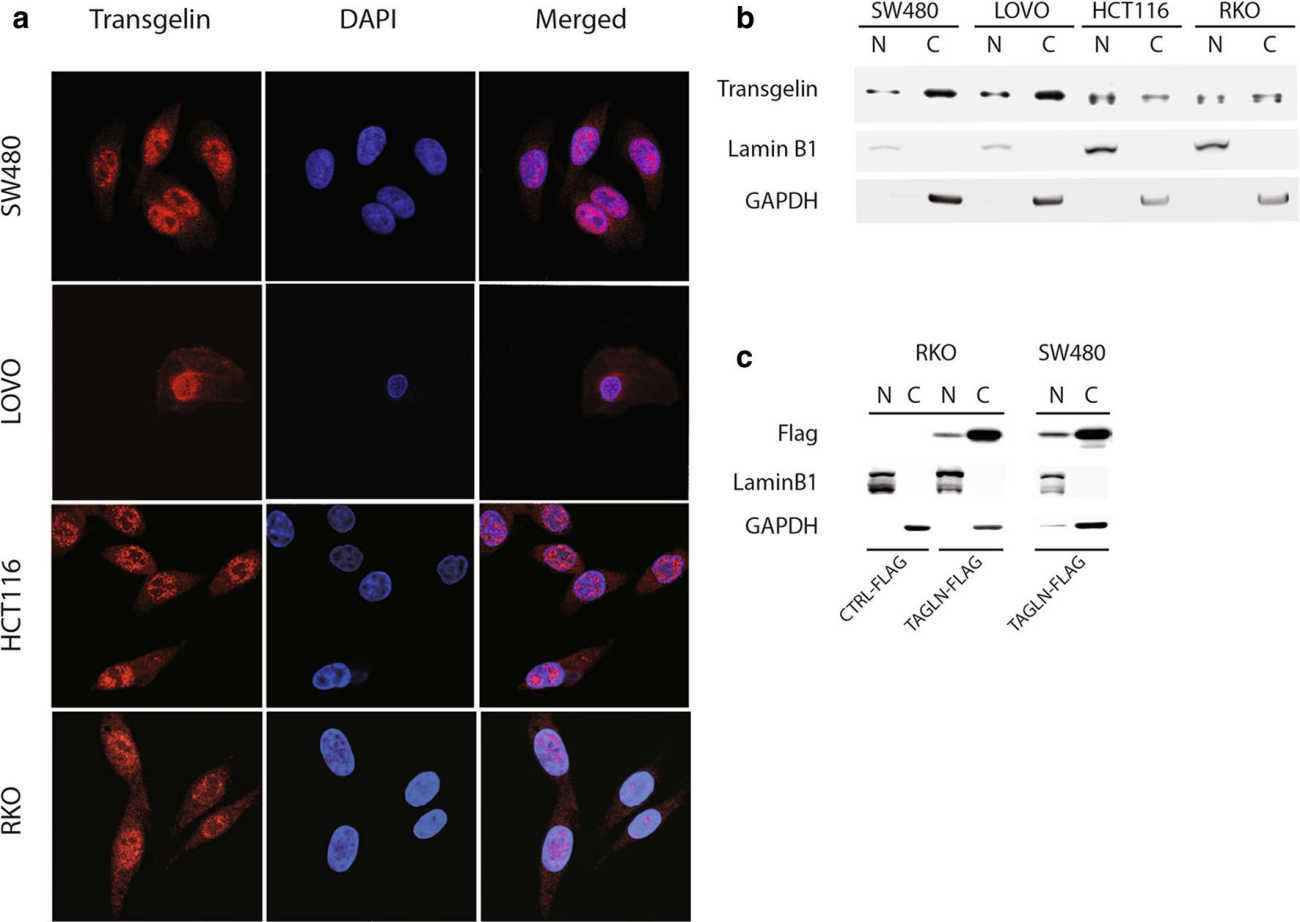
The localization of transgelin in different human colon cancer cell lines. **a** Transgelin in colon cancer cell lines RKO, SW480, HCT116 and LOVO was observed via immunofluorescence. The panels show transgelin immunostaining (red), 4′,6-diamidino-2-phenylindole DNA staining (DAPI), and a merged image is shown. **b** The distribution of transgelin in SW480, LOVO, HCT116 and RKO cells were identified via immunoblotting. **c** The distribution of transgelin-flag fusion protein in RKO and SW480 cells that were transiently transfected with pcDNA6/myc-His B-TAGLN-flag plasmid and control plasmid as detected via immunoblotting. N refers to the nuclear protein fraction, and C is the cytoplasmic protein fraction. Lamin B1 is a nuclear protein marker, GAPDH is a cytoplasmic protein marker

**Fig. 2:**
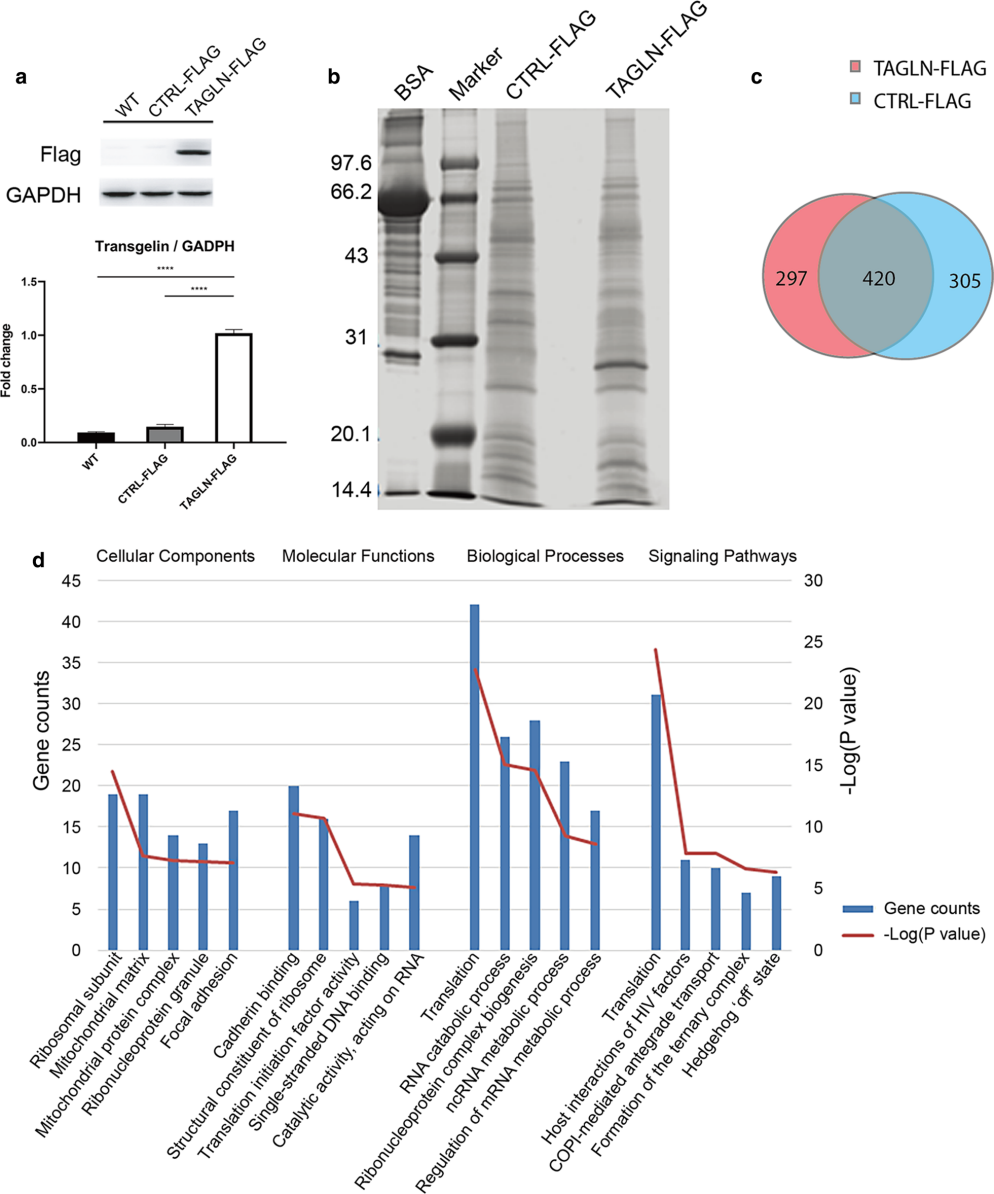
Identification of proteins potentially interacting with transgelin in the RKO cells. **a** Transgelin-flag protein was expressed in RKO cells transiently transfected with plasmids. Transgelin expression in RKO wild-type (WT), RKO-CTRL-FLAG and RKO-TAGLN-FLAG cells was detected via immunoblotting. **** P < 0.0001. **b** Proteins extracted from RKO-CTRL-FLAG and RKO-TAGLN-FLAG cells were immunoprecipitated with corresponding anti-flag antibody and visualized via silver staining. **c** The relationship between immunoprecipitated proteins by anti-flag antibody in RKO-CTRL-FLAG and RKO-TAGLN-FLAG cells. **d** Functional enrichment analysis of the 297 proteins potentially interacting with transgelin-flag fusion protein

The expression of transgelin in colon cancer cell lines (HCT116, SW480, RKO and LOVO) was detected by immunofluorescence and immunoblotting assays. Both cytoplasmic and nuclear localization of endogenous transgelin were observed (Fig. 1a, b). Further, pcDNA6/myc-His B-TAGLN-flag and pcDNA6/myc-His B-flag control plasmids were transiently transfected into RKO and SW480 cells. Immunoblotting analysis showed detectable levels of exogenous transgelin-flag protein in both the cytoplasm and nucleus (mainly in the cytoplasm) of the associated RKO and SW480 cells (Fig. 1c). The expression level of transgelin-flag protein (1.00 ± 0.05) was significantly increased in the RKO-TAGLN-FLAG cells compared with the RKO-CTRL-FLAG cells (0.13 ± 0.03, *P *< 0.0001, Fig. 2a) and the wild type (WT) RKO cells (0.08 ± 0.02, *P *< 0.0001).

### Identification of proteins potentially interacting with transgelin in the RKO cells

To explore the proteins that were potentially interacting with transgelin, we performed immunoprecipitation in the RKO-TAGLN-FLAG cells and the RKO-CTRL-FLAG cells using an anti-flag monoclonal antibody. We observed a clear band in the RKO-TAGLN-FLAG group, ranging from 20.1 to 31 kb in the silver staining gel (Fig. 2b). To identify the proteins in the samples, we performed the high-performance liquid chromatography coupled with the tandem mass spectrometry. Approximately 725 proteins were identified in the RKO-CTRL-FLAG group, while 717 were in the RKO-TAGLN-FLAG group (Additional files 2 and 3). However, about 297 proteins were uniquely present in the RKO-TAGLN-FLAG group (Fig. 2c, Additional file 4: Table S1). Gene ontology (GO) functional enrichment analysis suggested that the 297 proteins in the RKO-TAGLN-FLAG group were mainly involved in translation, RNA processing, enzyme activity and cell junction adherence (Fig. 2d). In addition, among these, 23 proteins were DNA-binding proteins (Table 1).

**Table 1:** DNA-binding proteins that were potentially interacted with Transgelin (FDR ≤ 0.01)

	gi number	Name of the protein	Molecular weight (Dalton)
1	gi|124494254	Proliferation-associated protein2G4	43,786
2	gi|114205460	HIST1H2BC protein	13,833
3	gi|21361745	Spermatid perinuclear RNA-binding protein	73,651
4	gi|4827071	Cellular nucleic acid-binding protein	19,462
5	gi|156523968	poly (ADP-ribose) polymerase family, member 1 (PARP1)	113,084
6	gi|29612542	Histone H2A	13,162
7	gi|6912616	Histone H2A	13,508
8	gi|323650782	HMGA2 fusion protein	13,811
9	gi|297262894	High mobility group protein HMGI-C	12,714
10	gi|4506491	Replication factor C subunit 4	36,877
11	gi|4502747	Cyclin-dependent kinase 9	42,777
12	gi|345783096	Barrier-to-autointegration factor	10,058
13	gi|7661672	Polymerase delta-interacting	42,032
14	gi|98986457	Host cell factor 1	208,730
15	gi|32129199	SAP domain-containing Ribonucleo protein	23,670
16	gi|57530065	CCR4-NOT transcription complex subunit 7	32,744
17	gi|302699237	Eukaryotic translation initiation factor 4 gamma 1	158,643
18	gi|5730027	KH domain-containing, RNA-binding, signal transduction-associated protein 1	48,226
19	gi|238066755	Disrupted in schizophrenia 1isoform 49	21,427
20	gi|351694577	Activated RNA polymerase II transcriptional coactivator p15	13,993
21	gi|119607091	DNA replication licensing factor MCM4	11,656
22	gi|7673373	SCAN-related protein RAZ1	23,430
23	gi|4758356	Flap endonuclease 1	42,592

### Effects of TAGLN over-expression on downstream genes and signaling pathways

**Fig. 3:**
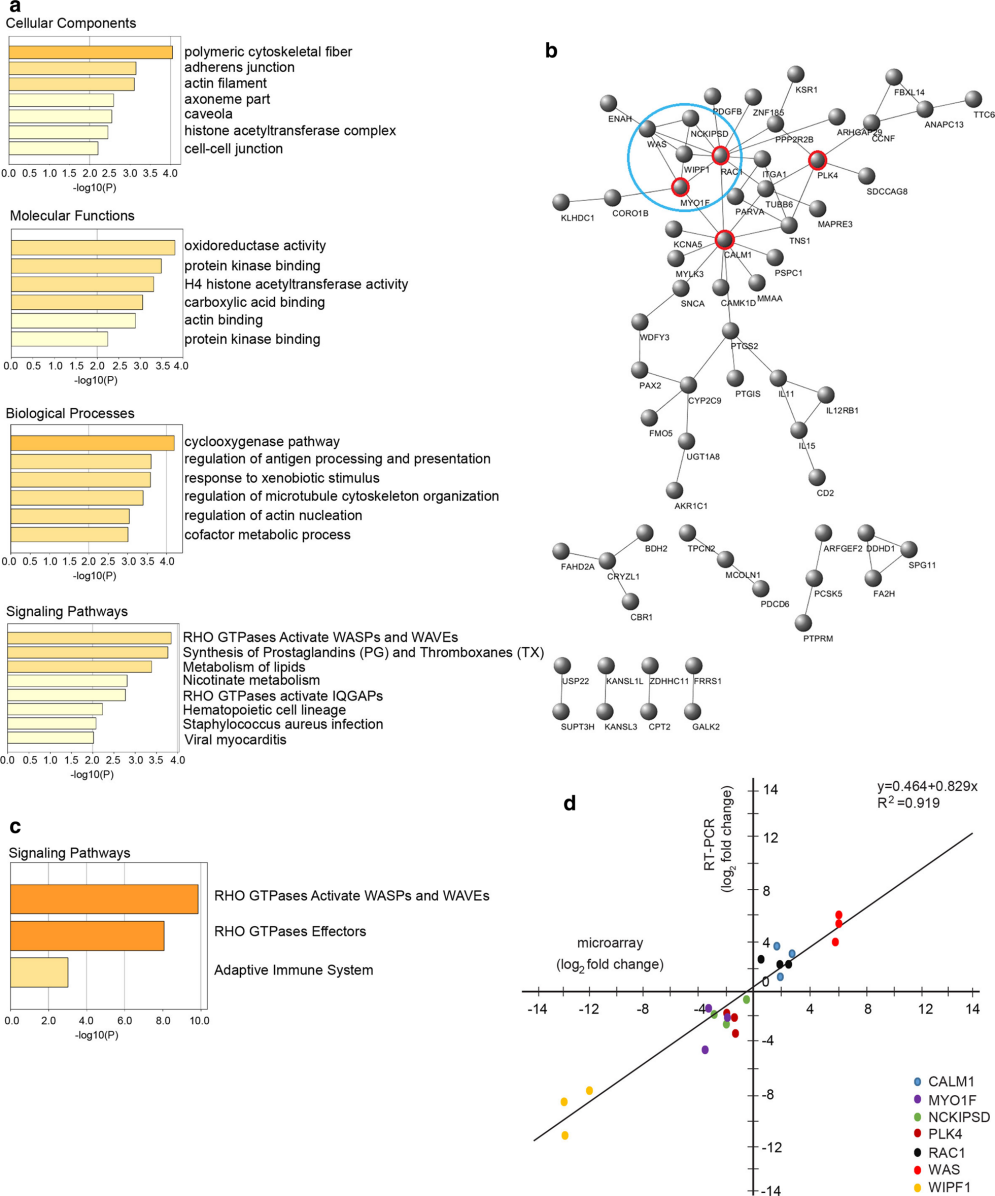
Effects of *TAGLN* overexpression on other genes and signaling pathways in RKO cells. Over-expression of *TAGLN* in RKO human colon cancer cells resulted in 184 genes differentially expressed with at least a twofold change (P < 0.05). **a** Functional enrichment (including cellular components, molecular functions, biological processes) and signaling pathway analysis of the DEGs were performed. **b** Topological analysis of the network illustrating the relationship between the proteins encoded by the DEGs. Genes in the red circle are core genes. The blue circle is the core module. The combinations of genes in the red and the blue circle are the key genes. **c** Signaling pathway enrichment analysis of the key genes identified the Rho signaling pathway. **d** Levels of the identified key genes as measured by cDNA microarray and real time RT-PCR. Relative gene expression was illustrated on a log-transformed scale. Data was obtained from three biological replicates

In our previous study, over-expression of *TAGLN* in RKO cells led to differential expression of the 256 transcripts in the Affymetrix cDNA microarray [9]. Of these, 68 transcripts with undefined gene symbols and gene database codes and 4 duplicates were eliminated. Finally, 184 DEGs (92 up-regulated and 92 down-regulated DEGs) were obtained for further analysis. Functional enrichment and signaling pathway enrichment analyses were performed using the Metascape tool (Fig. 3a). The results showed that the 184 DEGs were mainly associated with the cytoskeleton, protein kinase binding, regulation of cytoskeleton remodeling and Rho GTPase activation.

A PPI network was constructed with proteins encoded by the 184 DEGs using the STRING tool. The topological properties of the network were analyzed, which were composed of 167 nodes and 70 edges. The PPI network data were introduced into the Cytoscape (Fig. 3b). CytoHubba plug-ins were used to calculate the degree centrality and intermediate centrality of the DEGs. The mean values of global centrality and intermediate centrality were 4.375 and 153.375, respectively. Four genes, including *CALM1, RAC1, PLK4* and *MYO1F*, were selected as the core genes in this network (Fig. 3b in red circles). An MCODE plug-in was then utilized to analyze interactions within the network. A core module with 4.5 points was selected (Fig. 3b in blue circle) by the k kernel analysis (k = 2), consisting of five nodes (*RAC1, WAS, WIPF1, NCKIPSD, MYO1F*) and nine edges. Based on the STRING tool, there were also complex interactions among gene-encoded proteins.

The core genes and the genes included in the core module were combined and seven discrete genes, including *CALM1, PLK4, RAC1, WAS, WIPF1, NCKIPSD* and *MYO1F*, were selected as key genes. Signaling pathway enrichment analysis of the key genes was performed. Three entries, mainly involving the Rho GTPase signaling pathway with significant differences, were obtained (Fig. 3c). We validated the expression levels of the key genes using RNA from the RKO cells, which were used for microarray, by real time RT-PCR (Fig. 3d). A scatter plot illustrated the agreement between the cDNA microarray and RT-PCR, with a coefficient of determination, R^2^, of 0.919. The correlation was significant based on Pearson correlation analysis (P < 0.01).

### Prediction of the TF for the key genes and validation of its interaction with transgelin

**Fig. 4:**
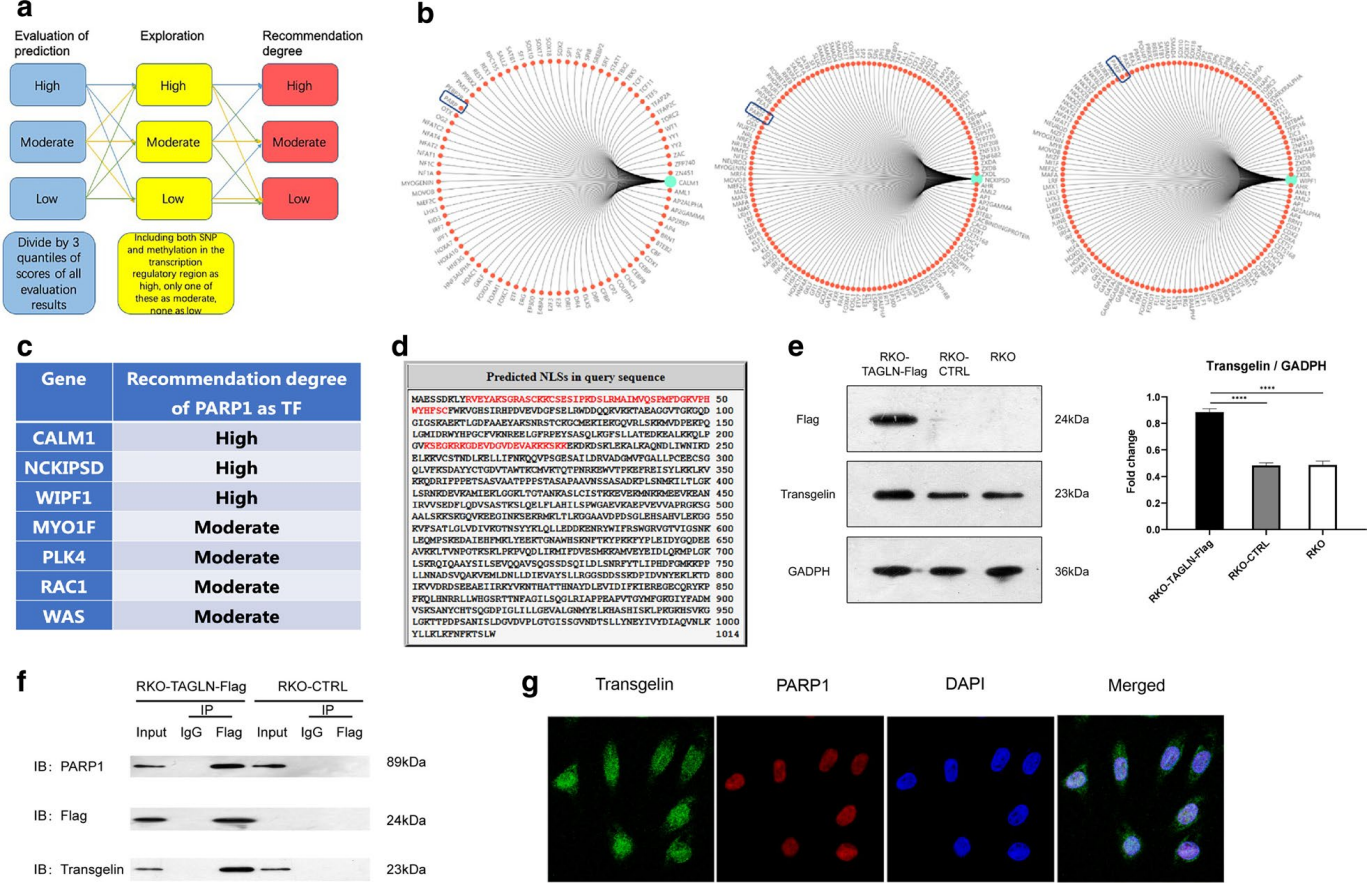
Prediction of the transcription factor(s) of the key genes and validation of transgelin-PARP1 interaction. **a** An illustration of the computational model for predicting the transcription factors of the key genes. **b** The prediction of the transcription factors of the key genes (partially illustrated). PARP1 protein is circled in a blue frame. **c** The recommendation degree of PARP1 as the transcription factor of the seven key genes downstream of transgelin. **d** The sequence of PARP1 protein. The red fonts represent the corresponding sequences of possible nuclear localization signal in PARP1 protein. **e** Immunoblotting analysis of transgelin and flag protein expression in RKO-TAGLN-FLAG, RKO-CTRL and wild type RKO cells, ****P < 0.0001. **f** Interaction between the transgelin-flag fusion protein and PARP1 was validated by co-immunoprecipitation. RKO-CTRL was the control group and normal rabbit IgG was used as the control antibody. **g** Immunofluorescence analysis of transgelin and PARP1 in RKO cells. The panels show transgelin immunostaining (green), PARP1 immunostaining (red), 4′,6-diamidino-2-phenylindole DNA staining (DAPI), and a merged image is indicated

We then analyzed the promoter regions of the seven key genes (*CALM1, PLK4, RAC1, WAS, WIPF1, NCKIPSD, MYO1F*) to explore if they share the same transcription factor(s) using the GCBI tools in Ensembl, Transfac, COSMIC and dbSNP databases. A computational model was utilized as described from the GCBI website (http://college.gcbi.com.cn/archives/2437) (Fig. 4a). PARP1 was predicted as the transcription factor for the seven key genes (Fig. 4b, c). It was also included in the 23 DNA-binding proteins potentially interacting with the transgelin-flag fusion protein (Table 1). Besides, the cNLS Mapper identified nuclear localization signals in the PARP1 protein (Fig. 4d).

To validate the interaction between PARP1 and transgelin, we transiently transfected pENTER-TAGLN-FLAG and pENTER-Flag control plasmids into the RKO cells. Immunoblotting analysis showed the over-expression of transgelin in RKO-TAGLN-FLAG cells as compared to the RKO-CTRL-FLAG cells (P < 0.0001, Fig. 4e). Immunoprecipitation followed by immunoblotting assays showed that the anti-flag antibody specifically immunoprecipitated PARP1 in the RKO-TAGLN-FLAG cells, validating its binding to the transgelin-flag fusion protein (Fig. 4f). In addition, immunofluorescence analysis indicated that endogenous transgelin was co-localized with PARP1 in the RKO cells (Fig. 4g).

## Discussion

### Transgelin in colon cancer metastasis

Transgelin is an actin-binding protein presumably existing in the cytoplasm of smooth muscle cells. Findings from our previous study showed that transgelin increased the metastatic potential of colon cancer cells by remodeling the cytoskeleton in the cytoplasm [10]; it also altered the expression of metastasis-related genes, thereby promoting the formation of metastatic phenotypes in the tumor cells [9]. Since many actin-binding proteins have been proven to exert different biological functions in the cytoplasm and nucleus [20–23], we hypothesized that transgelin could play a central role in the invasion and metastasis of colon cancer cells through specific mechanisms in different cellular localization.

In the present study, we found that both endogenous and exogenous transgelin were expressed in the cytoplasm and nucleus of the colon cancer cells (Fig. 1). In addition, transgelin was shown to interact with a variety of metabolic-related enzymes, transport proteins, transcription factors, and cytoskeletal proteins (Additional file 4: Table S1). These results indicate that transgelin is likely to have a nuclear-cytoplasmic shuttling and perform its biological functions in different cellular compartments that collaboratively participate in the invasion and metastasis of the colon cancer cells.

### Transgelin and Rho signaling pathway in colon cancer cells

At present, studies on actin and its interacting molecules largely focus on specific signaling pathways, such as the Rho GTPases and its downstream effector proteins, which mediate tumor cell migration, invasion and metastasis through cytoskeleton (reviewed in [24]).

Rho GTPase, a family of 20 small G proteins, interacts with downstream proteins to influence cell cycle, polarity, and migration by regulating the cytoskeleton [25]. In addition, various studies have suggested that an increase in the expression level of the Rho GTPase gene is associated with an increase in cell invasiveness and metastatic phenotype (reviewed in [24]). Rho GTPase interacts with Rho, Rac and Cdc42 in the eukaryotic cells to regulate the assembly and remodeling of the actin cytoskeleton (reviewed in [24]). Rho recruits Rho kinase (ROCK) and phosphorylates various cytoskeletal proteins, thus promoting the formation of actin fiber stress and generating contractile force [26, 27]. ROCK, a major downstream effector of the Rho GTPase family proteins, participates in the regulation of actin remodeling by phosphorylation of the cofilin and myosin light chain (MLC) [26, 27].

Based on the expression profiling data from our previous study [9], we obtained 184 DEGs and identified seven key genes, including *CALM1, MYO1F, NCKIPSD, PLK4, RAC1, WAS* and *WIPF1*, downstream of *TAGLN* using bioinformatics methods. These genes are associated with tumor formation and metastasis [28–36]. Moreover, they have been implicated in the Rho GTPases activation pathway, which could be a major pathway for transgelin to participate in colon cancer metastasis. Although some of the DEGs identified from the same cDNA microarray were validated by quantitative RT-PCR in another cell line (DLD-1) [9], the effects of transgelin on Rho signaling pathway warrant further investigation to fully uncover the underlying mechanisms.

### Transgelin interacts with PARP1 in colon cancer cells

Findings from this study confirmed the localization of transgelin in the nucleus of the colon cancer cells. Manipulation of transgelin expression resulted in differential expression of a variety of genes and affected the biological behaviors of the colon cancer cells in vitro and in vivo [9]. Although transgelin potentially interacted with 297 proteins, neither it bound directly to the RNA polymerase II (Additional file 4: Table S1) nor had a nuclear localization signal. Therefore, we speculate that transgelin may interact with other partner(s) to regulate the downstream target genes, thereby affecting colon cancer metastasis. After analyzing the promoter regions of the key genes downstream of *TAGLN* to predict their potential TF(s) (Fig. 4a–c) and comparing them with the 23 DNA-binding proteins that were interacting with transgelin (Table 1), PARP1 was found to be the only one mapping to both.

Poly (ADP-ribose) polymerase-1, a 113 kDa nuclear enzyme, is encoded by the *PARP1* gene [37]. Its N-terminal contains a DNA binding domain consisting of two zinc finger motifs and a nuclear localization sequence [37]. It is involved in DNA repair, cell cycle, cell death, tumorigenesis and other cellular processes [38–41]. PARP1 has been reported to play an important role in the early development and progression of CRC [42, 43]. It has also been found to promote tumor metastasis in soft tissue sarcoma [44] and non-small cell lung cancer [45]. Moreover, Dorsam et al. [46] have shown that PARP1 reduced the N-nitroso compounds (NOC)-induced tumorigenesis, regulated intestinal inflammation through innate immune response and promoted colorectal tumor growth. PARP1 has also been suggested to regulate the transcription of genes by directly binding to their promoters [47–49]. Taken together, these findings imply that PARP1 could be a promising target for malignant tumor intervention.

**Fig. 5:**
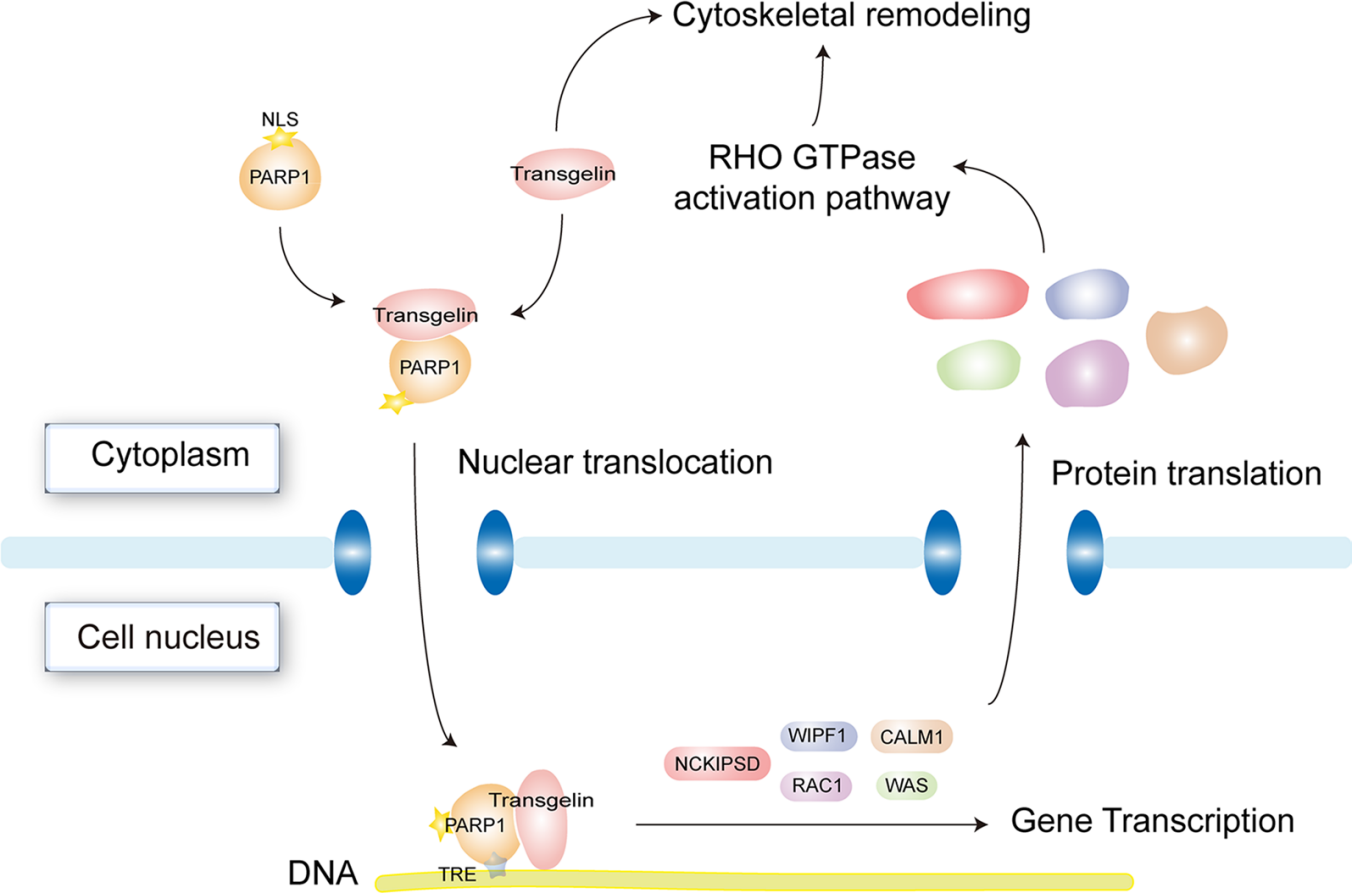
Proposed model of the transgelin mechanisms involved in promoting colon cancer metastasis. Once the cancer cells receive signals from the tumor micro-environment, transgelin directly remodels the cytoskeleton. It also binds to the PARP1 forming a complex which translocates into the nucleus where it regulates the expression of the key genes. Subsequently, the Rho signaling pathway is stimulated and initiates cytoskeletal remodeling which promotes colon cancer metastasis

In the current study, we validated the interaction between transgelin and PARP1 with immunoprecipitation and immunofluorescence assays (Fig. 4f, g). Although this study endeavors to delineate the mechanisms of how transgelin and PARP1 interaction influences the Rho signaling pathway and participates in colon cancer metastasis, a proper understanding of these mechanisms warrants more comprehensive analysis. We believe that transgelin functions through a dual mechanism. Firstly, we suppose that transgelin directly takes part in the cytoskeletal remodeling in the cytoplasm following cancer cells signaling from the tumor microenvironment. Secondly, transgelin binds to PARP1 forming a complex that translocates into the nucleus, where the complex regulates the expression of the key genes and subsequently affects the Rho GTPase activation pathway, initiating cytoskeletal remodeling (Fig. 5). The dual mechanism may simultaneously promote colon cancer metastasis.

## Conclusions

Our results support a hypothesis that transgelin interacts with PARP1 and regulates the expression of downstream key genes (*CALM1, MYO1F, NCKIPSD, PLK4, RAC1, WAS* and *WIPF1*), which are mainly involved in the Rho signaling pathway in the human RKO colon cancer cells.

## Supplementary information

**Additional file 1: Table S2.** PCR primers of the identified key genes.

**Additional file 2.** The metadata of protein identification with high-performance liquid chromatography/tandem mass spectrometry. The dataset included two sheets; the NC sheet contained proteins identified in the RKO-CTRL-FLAG group, while the TAGLN sheet contained proteins identified in the RKO-TAGLN-FLAG group.

**Additional file 3.** The metadata of peptide identified with high-performance liquid chromatography/tandem mass spectrometry. The dataset included two sheets; the NC sheet contained peptides identified in the RKO-CTRL-FLAG group, while the TAGLN sheet contained peptides identified in the RKO-TAGLN-FLAG group.

**Additional file 4: Table S1.** Proteins potentially interacting with a transgelin-flag fusion protein (FDR ≤ 0.01). Proteins that were uniquely present in the RKO-TAGLN-FLAG group were listed after the exclusion of those present both in the RKO-CTRL-FLAG and RKO-TAGLN-FLAG groups.

## Data Availability

The microarray datasets analyzed in this manuscript have been deposited in NCBI’s Gene Expression Omnibus and are accessible through GEO Series accession number GSE48998 (https://www.ncbi.nlm.nih.gov/geo/query/acc.cgi?acc=GSE48998). Other datasets generated and/or analyzed during the current study are included within the article and its additional files.

## Abbreviations

CRC: Colorectal cancer
GCBI: Gene-cloud of biotechnology and information
TF: Transcription factor
PARP1: Poly ADP-ribose polymerase-1
CH: Calmodulin homologous
CLIK: C-terminal calmodulin like
TGF β: Transforming growth factor β
EMT: Epithelial to mesenchymal transition
MEM: Minimum Eagle’s medium
RPMI: Roswell Park Memorial Institute
DAPI: 4′,6-diamidino-2-phenylindole
RT-PCR: Reverse transcription-polymerase chain reaction
HRP: Horseradish peroxidase
FASP: Filtered aided proteome preparation
FDR: False discovery rate
DEGs: Differential expression genes
GO: Gene ontology
KEGG: Kyoto Encyclopedia of Genes and Genomes
PPI: Protein–protein interaction
NLS: Nuclear localization signal
SD: Standard deviation
WT: Wild type
ROCK: Rho kinase
MLC: Myosin light chain
NOC: Nitroso compound
