# Supplementary material for: Transgelin interacts with PARP1 in human colon cancer cells
Source: Cancer Cell Int. 2020 Aug 3;20:366. doi: 10.1186/s12935-020-01461-y (PMC7398379; doi:10.1186/s12935-020-01461-y)
Supplement: Supplementary file 1 — Additional file 1: Table S2. PCR primers of the identified key genes. [file 12935_2020_1461_MOESM1_ESM.docx]

Supplementary Table 2. PCR primers of the identified key genes.

| Genes | 5’ to 3’ | 3’ to 5’ |
| --- | --- | --- |
| CALM1 | CTTCCAGTCCGCAGAGAGATG | GCCAAACCAGAAACGCAGTC |
| MYO1F | CCAATCGGACACCTACCAGG | CTGGGCGGGATCCCAATAAC |
| NCKIPSD | CTTCCCAGATCCCACCACAG | CTGGTTCAGATGGGCTGGAG |
| PLK4 | AAGCTCGACACTTCATGCACC | GCATTTTCAGTTGAGTTGCCAG |
| RAC1 | ATGTCCGTGCAAAGTGGTATC | CTCGGATCGCTTCGTCAAACA |
| WAS | TGACATCACGAGTTCACGATACC | GTGGCTGACATGCTTGAATCC |
| WIPF1 | ACGGCCAACAGGGATAATGAT | GGTTTCGCAGATGTGGATCTT |
