# Supplementary material for: Transgelin interacts with PARP1 in human colon cancer cells
Source: Cancer Cell Int. 2020 Aug 3;20:366. doi: 10.1186/s12935-020-01461-y (PMC7398379; doi:10.1186/s12935-020-01461-y)
Supplement: Supplementary file 4 — Additional file 4: Table S1. Proteins potentially interacting with a transgelin-flag fusion protein (FDR ≤ 0.01). Proteins that were uniquely present in the RKO-TAGLN-FLAG group were listed after the exclusion of those present both in the RKO-CTRL-FLAG and RKO-TAGLN-FLAG groups. [file 12935_2020_1461_MOESM4_ESM.docx]

Supplementary Table 1. Proteins that were potentially interacted with Transgelin-flag fusion protein (FDR≤0.01).

| No. | Uniprot  Entry | Gi number | Uniprot_Protein name |
| --- | --- | --- | --- |
| 1 | B4DE78 | 296192282 | 14-3-3 protein gamma-like isoform 2 |
| 2 | H0YJX2 | 149622137 | 26S protease regulatory subunit 10B-like, Partial |
| 3 | B4DP77 | 16554607 | 28S ribosomal protein S10, Mitochondrial |
| 4 | J3QLR8 | 114669508 | 28S ribosomal protein S23, Mitochondrial isoform 2 |
| 5 | Q92552 | 186928850 | 28S ribosomal protein S27, Mitochondrial |
| 6 | Q92665 | 186928854 | 28S ribosomal protein S31, Mitochondrial precursor |
| 7 | H0YG82 | 16950603 | 28S ribosomal protein S35, Mitochondrial isoform 1  precursor |
| 8 | Q86WV4 | 33188463 | 28S ribosomal protein S9, Mitochondrial precursor |
| 9 | B7Z358 | 302563543 | 39S ribosomal protein L50, Mitochondrial |
| 10 | Q99714 | 4758504 | 3-hydroxyacyl-CoA dehydrogenase type-2 isoform 1 |
| 11 | Q71UM5 | 109104395 | 40S ribosomal protein S27-like |
| 12 | B4E3C2 | 297702579 | 60S ribosomal protein L17-like |
| 13 | Q9BQQ5 | 4506625 | 60S ribosomal protein L27a |
| 14 | K7ELC7 | 348562769 | 60S ribosomal protein L27-like |
| 15 | B2R4V2 | 344306874 | 60S ribosomal protein L36a-like |
| 16 | Q9Y3U8 | 296232623 | 60S ribosomal protein L36-like |
| 17 | C9J4Z3 | 344268217 | 60S ribosomal protein L37a-like |
| 18 | D6RAN4 | 15431303 | 60S ribosomal protein L9 |
| 19 | B1APP6 | 11321601 | 6-phosphofructokinase type C isoform 1 |
| 20 | P53396 | 68533125 | ACLY variant protein |
| 21 | D6R970 | 351694577 | Activated RNA polymerase II transcriptional  coactivator p15 |
| 22 | B2R4I8 | 119569337 | Adaptor-related protein complex 3, Sigma 1 subunit,  Isoform CRA_a |
| 23 | P27144 | 8051579 | Adenylate kinase isoenzyme 4, Mitochondrial  isoform 6 |
| 24 | B7ZB63 | 297262255 | ADP-ribosylation factor 3-like isoform 3 |
| 25 | B4DR45 | 109148542 | Alanyl-tRNA synthetase, Cytoplasmic |
| 26 | P54920 | 47933379 | Alpha-soluble NSF attachment protein |
| 27 | J7KF88 | 14149904 | Anthrax toxin receptor 1 isoform 1 precursor |
| 28 | Q53ZP9 | 4579911 | Apg-1 |
| 29 | Q59GY0 | 62087538 | Apolipoprotein B mRNA editing enzyme, Catalytic  polypeptide-like 3C variant |
| 30 | B4E283 | 219283152 | Apoptosis inhibitor 5 isoform a |
| 31 | Q9BVC5 | 13129094 | Ashwin |
| 32 | H3BSK9 | 354498028 | Ataxin-2-like protein |
| 33 | P30049 | 4502297 | ATP synthase subunit delta, Mitochondrial precursor |
| 34 | O95817 | 14043024 | BAG family molecular chaperone regulator 3 |
| 35 | O75531 | 345783096 | Barrier-to-autointegration factor isoform 1 |
| 36 | B1AH87 | 34576920 | Benzodiazapine receptor (peripheral) |
| 37 | P07814 | 62241042 | Bifunctional aminoacyl-tRNA synthetase |
| 38 | Q8WY22 | 19923665 | BRI3-binding protein precursor |
| 39 | Q9HDC9 | 9836652 | BSCv |
| 40 | G3V3L6 | 222136639 | C-1-tetrahydrofolate synthase, Cytoplasmic |
| 41 | B4E1R7 | 297661901 | Calpain-2 catalytic subunit-like isoform 2 |
| 42 | B4DNA3 | 55859737 | CAP, Adenylate cyclase-associated protein 1 (yeast) |
| 43 | K7ERZ3 | 3095186 | Cargo selection protein TIP47 |
| 44 | B4E1D9 | 68303572 | Casein kinase I isoform alpha isoform 2 |
| 45 | H3BNI9 | 6753540 | Casein kinase II subunit alpha' |
| 46 | B7Z6J7 | 332857277 | CCR4-NOT transcription complex subunit 3 isoform 1 |
| 47 | Q9UIV1 | 57530065 | CCR4-NOT transcription complex subunit 7 |
| 48 | Q7Z759 | 31418053 | CCT8 protein |
| 49 | B4DEZ3 | 12005918 | CDA016 |
| 50 | B4DDL9 | 297692162 | Cell division protein kinase 2-like isoform 3 |
| 51 | Q4JGY1 | 157909782 | Cellular nucleic acid-binding protein isoform 2 |
| 52 | P62633 | 4827071 | Cellular nucleic acid-binding protein isoform 3 |
| 53 | O00299 | 14251209 | Chloride intracellular channel protein 1 |
| 54 | E9PLD3 | 119594460 | Chromosome 11 open reading frame 48, Isoform  CRA_c |
| 55 | Q8WWC4 | 17389900 | Chromosome 2 open reading frame 47 |
| 56 | Q3YEC7 | 55958747 | Chromosome 9 open reading frame 86 |
| 57 | A6NDF3 | 119619028 | Chromosome X open reading frame 26, Isoform  CRA_b |
| 58 | O75390 | 38327625 | Citrate synthase, Mitochondrial precursor |
| 59 | B3KVX6 | 63102283 | CKAP4 protein |
| 60 | P53618 | 7705369 | Coatomer subunit beta |
| 61 | K7EPV0 | 94536771 | Coiled-coil domain-containing protein 56 |
| 62 | Q9Y2S6 | 7705431 | Coiled-coil domain-containing protein 72 |
| 63 | Q9UBW8 | 7705330 | COP9 signalosome complex subunit 7a |
| 64 | Q53FV3 | 62897917 | COP9 signalosome subunit 4 variant |
| 65 | P22528 | 83582815 | Cornifin-B |
| 66 | Q53G58 | 62897707 | Coronin, Actin binding protein, 1C variant |
| 67 | B7Z9C4 | 332248480 | CTP synthase 1 |
| 68 | Q13616 | 32307161 | Cullin-1 |
| 69 | P50750 | 4502747 | Cyclin-dependent kinase 9 |
| 70 | P01037 | 19882251 | Cystatin-SN precursor |
| 71 | B4DXK9 | 312222769 | Cysteine desulfurase, Mitochondrial isoform b  precursor |
| 72 | O14949 | 83367083 | Cytochrome b-c1 complex subunit 8 |
| 73 | F8W8S0 | 24307879 | Cytoplasmic dynein 1 intermediate chain 2 |
| 74 | B4DZP4 | 296231281 | Cytoplasmic dynein 1 light intermediate chain 2-like  isoform 2 |
| 75 | Q96EP5 | 25470886 | DAZ-associated protein 1 isoform b |
| 76 | H7BXY3 | 18043040 | DEAH (Asp-Glu-Ala-His) box polypeptide 30 |
| 77 | P33316 | 70906441 | Deoxyuridine 5'-triphosphate nucleotidohydrolase , Mitochondrial isoform 1 precursor |
| 78 | B7Z845 | 30506 | Desmoglein type 1 |
| 79 | C4P0D4 | 238066751 | Disrupted in schizophrenia 1 isoform 47 |
| 80 | C9JBJ6 | 332214985 | DNA-directed RNA polymerases I, II, And III subunit  RPABC3-like isoform 1 |
| 81 | C9JUL4 | 62089430 | DnaJ (Hsp40) homolog, Subfamily B, Member 4 variant |
| 82 | E9PNQ1 | 297269559 | Dolichyl-diphosphooligosaccharide--protein  glycosyltransferase subunit STT3A-like isoform 4 |
| 83 | E9PHH5 | 56203022 | Dolichyl-phosphate mannosyltransferase polypeptide  1, Catalytic subunit |
| 84 | Q13561 | 22096346 | Dynactin subunit 2 |
| 85 | Q8TE73 | 19115954 | Dynein heavy chain 5, Axonemal |
| 86 | F8W1G0 | 119618398 | Endoplasmic reticulum protein 29, Isoform CRA_b |
| 87 | O43324 | 4758862 | Eukaryotic translation elongation factor 1 epsilon-1  isoform 1 |
| 88 | P47813 | 301756272 | Eukaryotic translation initiation factor 1A,  X-chromosomal-like |
| 89 | H3BPE3 | 332863578 | Eukaryotic translation initiation factor 3 subunit C-like |
| 90 | B3KW56 | 296227373 | Eukaryotic translation initiation factor 3 subunit E |
| 91 | B7ZAM9 | 10801345 | Eukaryotic translation initiation factor 3 subunit K |
| 92 | C9K0Q7 | 321173836 | Eukaryotic translation initiation factor 3, Subunit E  interacting protein |
| 93 | B2RU06 | 168270862 | Eukaryotic translation initiation factor 4 gamma 1 |
| 94 | Q5RKV6 | 17402904 | Exosome complex component MTR3 |
| 95 | E9PI41 | 351713983 | Exosome complex exonuclease RRP41 |
| 96 | O14980 | 4507943 | Exportin-1 |
| 97 | B4DPS6 | 332207749 | Exportin-2-like |
| 98 | B4DV73 | 154355000 | Far upstream element-binding protein 2 |
| 99 | Q59FU3 | 62088312 | Far upstream element-binding protein variant |
| 100 | E9PCI9 | 209571584 | Farnesyl pyrophosphate synthase isoform b |
| 101 | Q68CT4 | 114587555 | Filamin-B isoform 10 |
| 102 | I3L3E9 | 4758356 | Flap endonuclease 1 |
| 103 | Q14254 | 94538362 | Flotillin-2 |
| 104 | I0CE67 | 109104062 | Four and a half LIM domains protein 2-like isoform 8 |
| 105 | Q9Y5B6 | 22035565 | GC-rich sequence DNA-binding factor 1 isoform 1 |
| 106 | B4DZW9 | 332831319 | GDP-L-fucose synthase isoform 5 |
| 107 | A8K9K4 | 62020522 | Glucosidase I |
| 108 | B3KT18 | 297714329 | Glutamate dehydrogenase 1, Mitochondrial-like  isoform 1 |
| 109 | P35754 | 4504025 | Glutaredoxin-1 |
| 110 | B2C310 | 2204207 | Glutathione S-transferase |
| 111 | P06737 | 71037379 | Glycogen phosphorylase, Liver form isoform 1 |
| 112 | D6REM1 | 119631186 | Golgi phosphoprotein 3 (coat-protein), Isoform CRA_b |
| 113 | Q99988 | 153792495 | Growth/differentiation factor 15 precursor |
| 114 | D6RAC2 | 345329725 | Guanine nucleotide-binding protein subunit  beta-2-like 1-like |
| 115 | C9J0D1 | 29612542 | H2afvl protein |
| 116 | Q2VIR3 | 119616598 | HCG18050 |
| 117 | F8WE39 | 119584402 | HCG1995701, Isoform CRA_a |
| 118 | B7ZW15 | 119618140 | HCG2015269, Isoform CRA_c |
| 119 | Q6ZQN2 | 119594427 | HCG2020155, Isoform CRA_b |
| 120 | Q8N0T1 | 119607530 | HCG20884, Isoform CRA_a |
| 121 | P51858 | 4758516 | Hepatoma-derived growth factor isoform a |
| 122 | Q5SWC8 | 55961948 | Heterochromatin protein 1, Binding protein 3 |
| 123 | B4DTA2 | 149521754 | Heterogeneous nuclear ribonucleoprotein D-like  isoform 1 |
| 124 | Q96E39 | 21361809 | Heterogeneous nuclear ribonucleoprotein G-like 1 |
| 125 | B3KRA9 | 184021 | Hexokinase 1 |
| 126 | Q0D2M2 | 114205460 | HIST1H2BC protein |
| 127 | F5H6H0 | 52352850 | HMGA2e' |
| 128 | P51610 | 98986457 | Host cell factor 1 |
| 129 | B7Z909 | 5453832 | Hypoxia up-regulated protein 1 precursor |
| 130 | Q6ZVX0 | 41351320 | IGH@ protein |
| 131 | C9J4U1 | 348556802 | Importin subunit alpha-1-like |
| 132 | B4E0R6 | 4033763 | Importin-5 |
| 133 | E5RIP7 | 332828965 | Inositol monophosphatase 1-like isoform 4 |
| 134 | P09914 | 116534937 | Interferon-induced protein with tetratricopeptide  repeats 1 isoform 2 |
| 135 | Q5T765 | 31542980 | Interferon-induced protein with tetratricopeptide  repeats 3 |
| 136 | P48735 | 28178832 | Isocitrate dehydrogenase [NADP], Mitochondrial  precursor |
| 137 | B4DDX2 | 296207328 | KH domain-containing, RNA-binding, Signal  transduction-associated protein 1-like isoform 3 |
| 138 | O94925 | 40788380 | KIAA0838 protein |
| 139 | Q7Z434 | 6331231 | KIAA1271 protein |
| 140 | Q13601 | 117676403 | KRR1 small subunit processome component homolog |
| 141 | Q96RS2 | 14583014 | Laminin receptor-like protein LAMRL5 |
| 142 | B2R602 | 119590894 | LanC lantibiotic synthetase component C-like 1  (bacterial), Isoform CRA_b |
| 143 | B4E1Q7 | 400668 | Lipoamide acyltransferase component of  branched-chain alpha-keto acid dehydrogenase complex, Mitochondrial |
| 144 | B4DH39 | 332861443 | Long-chain-fatty-acid--CoA ligase 4 isoform 3 |
| 145 | P13473 | 4504957 | Lysosome-associated membrane glycoprotein 2  isoform A precursor |
| 146 | Q15046 | 5031815 | Lysyl-tRNA synthetase isoform 2 |
| 147 | Q4QZC0 | 123208811 | Major histocompatibility complex, Class I, A |
| 148 | Q5RZA0 | 21952531 | Mammary apoptosis inducing factor |
| 149 | E9PGM5 | 29387211 | MAP4 protein |
| 150 | B4DF61 | 15929104 | MARS protein |
| 151 | G3V117 | 148676796 | MCG145920, Isoform CRA_b |
| 152 | P62891 | 293356769 | MCG146274-like |
| 153 | H0YN88 | 148675001 | MCG15301, Isoform CRA_b |
| 154 | E5RK04 | 119607091 | MCM4 minichromosome maintenance deficient 4  (S. Cerevisiae), Isoform CRA_b |
| 155 | F6KRM6 | 333069620 | MHC class I antigen |
| 156 | Q8N183 | 29789409 | Mimitin, Mitochondrial |
| 157 | F8W0A9 | 62898307 | Mitochondrial aldehyde dehydrogenase 2 precursor  variant |
| 158 | B4DQY2 | 332813693 | Mitochondrial inner membrane protein |
| 159 | P14649 | 4505303 | Myosin light chain 6B |
| 160 | Q53FM7 | 62898071 | NADH dehydrogenase (ubiquinone) Fe-S protein 3,  30kDa (NADH-coenzyme Q reductase) variant |
| 161 | Q5H9R2 | 197101299 | NADH dehydrogenase [ubiquinone] 1 alpha  subcomplex subunit 5 |
| 162 | Q9BU61 | 41327781 | NADH dehydrogenase [ubiquinone] 1 alpha subcomplex  assembly factor 3 isoform a |
| 163 | K7ELZ9 | 119589740 | Nicalin homolog (zebrafish), Isoform CRA_d |
| 164 | Q5JPE7 | 51944971 | Nodal modulator 2 isoform 1 precursor |
| 165 | B7ZAC7 | 255683293 | Nucleosome assembly protein 1-like 4b |
| 166 | G3V3M5 | 148670802 | Numb gene homolog (Drosophila), Isoform CRA_b |
| 167 | Q53FF5 | 62898229 | P47 protein isoform a variant |
| 168 | Q6P1J9 | 22122445 | Parafibromin |
| 169 | Q02790 | 4503729 | Peptidyl-prolyl cis-trans isomerase FKBP4 |
| 170 | B7ZVW3 | 32455262 | Peroxiredoxin-5, Mitochondrial isoform c precursor |
| 171 | B4DNV1 | 313482810 | Peroxisomal multifunctional enzyme type 2 isoform 1 |
| 172 | P13796 | 167614506 | Plastin-2 |
| 173 | B4E0E1 | 297280655 | Poly [ADP-ribose] polymerase 1 isoform 1 |
| 174 | F8VXH9 | 354490197 | Poly(rC)-binding protein 2 isoform 4 |
| 175 | B4DEM9 | 332256102 | Polymerase delta-interacting protein 2 isoform 2 |
| 176 | Q8TCS8 | 188528628 | Polyribonucleotide nucleotidyltransferase 1,  Mitochondrial precursor |
| 177 | Q9Y3B4 | 7706326 | Pre-mRNA branch site protein p14 |
| 178 | Q9H000 | 32880199 | Probable E3 ubiquitin-protein ligase makorin-2 |
| 179 | K7EJ44 | 119610788 | Profilin 1, Isoform CRA_b |
| 180 | P12273 | 4505821 | Prolactin-inducible protein precursor |
| 181 | Q9UQ80 | 124494254 | Proliferation-associated protein 2G4 |
| 182 | C9J502 | 262070596 | Prostate leucine zipper variant 2 |
| 183 | Q16186 | 28373192 | Proteasomal ubiquitin receptor ADRM1 precursor |
| 184 | B4DJ66 | 119581642 | Proteasome (prosome, Macropain) 26S subunit,  Non-ATPase, 13, Isoform CRA_d |
| 185 | Q5JXJ1 | 56204043 | Proteasome (prosome, Macropain) subunit, Alpha  type, 7 |
| 186 | H0YNE3 | 30581141 | Proteasome activator complex subunit 1 isoform 2 |
| 187 | F5H169 | 2055256 | Proteasome subunit p27 |
| 188 | B4DNL5 | 197101103 | Protein disulfide-isomerase precursor |
| 189 | K7EN27 | 114552471 | Protein DJ-1 isoform 6 |
| 190 | B0AZT6 | 114577935 | Protein FAM136A-like isoform 1 |
| 191 | Q6FIA3 | 296841095 | Protein kinase C and casein kinase substrate in  neurons protein 2 isoform B |
| 192 | I3L4Q1 | 327290070 | Protein LSM14 homolog A-like isoform 2 |
| 193 | E5RJU9 | 223555917 | Protein LYRIC |
| 194 | B4DQI7 | 321267511 | Protein NipSnap homolog 1 isoform 2 |
| 195 | H0YHI8 | 351715284 | Protein phosphatase 1 regulatory subunit 12A |
| 196 | G3XAL7 | 18677735 | Protein PRRC1 |
| 197 | Q5VXV3 | 170763500 | Protein SET isoform 1 |
| 198 | Q05BX4 | 33875930 | PSMD1 protein |
| 199 | H0YGV8 | 48145661 | PSMD3 |
| 200 | J3QR88 | 345329219 | Pyrroline-5-carboxylate reductase 1,  Mitochondrial-like, Partial |
| 201 | B4DRT3 | 332164781 | Pyruvate kinase isozymes M1/M2 isoform f |
| 202 | C9JH92 | 13236495 | Quinone oxidoreductase isoform a |
| 203 | P50395 | 6598323 | Rab GDP dissociation inhibitor beta isoform 1 |
| 204 | Q53EY3 | 62898744 | RAN binding protein 1 variant |
| 205 | Q53HH4 | 62896771 | Ras-GTPase-activating protein SH3-domain-binding  protein variant |
| 206 | Q53T70 | 7710086 | Ras-related protein Rab-10 |
| 207 | P61106 | 18390323 | Ras-related protein Rab-14 |
| 208 | H7C125 | 326917676 | Ras-related protein Rab-2A-like |
| 209 | F5H157 | 5803135 | Ras-related protein Rab-35 isoform 1 |
| 210 | B4DEK7 | 348556994 | Ras-related protein Rab-8A-like |
| 211 | H0YJU2 | 149025275 | RCG20659, Isoform CRA_c |
| 212 | H7C367 | 149042187 | RCG36231, Isoform CRA_c |
| 213 | Q5T1D1 | 62511064 | RecName: Full=60S ribosomal protein L29 |
| 214 | Q5U0C2 | 54696204 | Replication factor C (activator 1) 3, 38kDa |
| 215 | P35249 | 4506491 | Replication factor C subunit 4 |
| 216 | P35244 | 4506587 | Replication protein A 14 kDa subunit |
| 217 | Q53R94 | 24431935 | Reticulon-4 isoform A |
| 218 | E9PIK5 | 190848 | Ribonuclease/angiogenin inhibitor |
| 219 | B2R6T7 | 4506127 | Ribose-phosphate pyrophosphokinase 1 isoform 1 |
| 220 | Q6IPH7 | 17932938 | Ribosomal protein L14 |
| 221 | G5E9L2 | 62088834 | Ribosomal protein L28 variant |
| 222 | F8VZ45 | 18088374 | Ribosomal protein L6 |
| 223 | Q5T8U2 | 55958184 | Ribosomal protein L7a |
| 224 | P62877 | 297261146 | RING-box protein 1-like |
| 225 | O00442 | 4506589 | RNA 3'-terminal phosphate cyclase isoform b |
| 226 | Q59GV2 | 62087594 | RNA binding motif protein 14 variant |
| 227 | E9PB51 | 297267337 | RNA-binding protein 4 isoform 5 |
| 228 | H7BY36 | 74007858 | RNA-binding protein EWS-like isoform 3 |
| 229 | Q6FG99 | 49457412 | RPLP1 |
| 230 | Q6IPX4 | 47940482 | RPS16 protein |
| 231 | Q9Y3Z3 | 38016914 | SAM domain and HD domain-containing protein 1 |
| 232 | P82979 | 32129199 | SAP domain-containing ribonucleoprotein |
| 233 | H7C5W9 | 296212897 | Sarcoplasmic/endoplasmic reticulum calcium ATPase  2 isoform 1 |
| 234 | H0UIA5 | 319655725 | SCAN domain-containing protein 1 isoform 2 |
| 235 | Q14162 | 33598929 | Scavenger receptor class F member 1 isoform 1  precursor |
| 236 | Q8IW48 | 26996830 | SDHA protein |
| 237 | Q92997 | 41406097 | Segment polarity protein dishevelled homolog DVL-3 |
| 238 | Q5HYG8 | 261862348 | Serine hydroxymethyltransferase, Mitochondrial  isoform 3 |
| 239 | B4DN87 | 296217036 | Serpin H1 isoform 2 |
| 240 | Q53GC9 | 297269274 | SID1 transmembrane family member 2 isoform 2 |
| 241 | Q5U0D2 | 297269274 | SID1 transmembrane family member 2 isoform 2 |
| 242 | G5E9Z8 | 119625903 | Signal recognition particle 72kDa, Isoform CRA_c |
| 243 | Q8WVW9 | 4507217 | Signal recognition particle 9 kDa protein isoform 2 |
| 244 | O95036 | 4176369 | Similar to 60S ribosomal protein L7; similar to P18124  (PID:d133021) |
| 245 | Q9H5Z5 | 19264156 | Similar to DKFZP434N178 protein |
| 246 | A4D198 | 51094971 | Similar to mKIAA0038 protein |
| 247 | K7EJB5 | 345315929 | Small nuclear ribonucleoprotein Sm D2-like |
| 248 | Q5XPV6 | 354473769 | Small nuclear ribonucleoprotein-associated protein  B-like |
| 249 | Q15515 | 804765 | Small proline-rich protein |
| 250 | B4E229 | 333360858 | Smoothelin isoform d |
| 251 | Q13813 | 62089306 | Spectrin, Alpha, Non-erythrocytic 1 (alpha-fodrin)  variant |
| 252 | D6W5C0 | 119620543 | Spectrin, Beta, Non-erythrocytic 1, Isoform CRA_b |
| 253 | Q96SI9 | 21361745 | Spermatid perinuclear RNA-binding protein isoform 1 |
| 254 | P63208 | 332821941 | S-phase kinase-associated protein 1-like isoform 5 |
| 255 | Q12874 | 5803167 | Splicing factor 3A subunit 3 |
| 256 | B4DUA4 | 149025035 | Splicing factor, Arginine/serine-rich 5, Isoform CRA_c |
| 257 | B7Z4N6 | 297299640 | Stathmin-2-like |
| 258 | Q5TCU6 | 55859707 | Talin 1 |
| 259 | B7Z8B1 | 296223758 | T-complex protein 1 subunit delta isoform 2 |
| 260 | K7EJM5 | 130750552 | TDP43 |
| 261 | Q6DKK2 | 332278244 | Tetratricopeptide repeat protein 19, Mitochondrial |
| 262 | A8K4K9 | 7661910 | Tetratricopeptide repeat protein 35 |
| 263 | Q9P016 | 7661804 | Thymocyte nuclear protein 1 isoform 1 |
| 264 | Q96B58 | 62088002 | TIA1 protein variant |
| 265 | Q9BVV7 | 156142176 | TIM21-like protein, Mitochondrial precursor |
| 266 | F8WBG6 | 21707652 | TM4SF1 protein |
| 267 | E9PKD1 | 168984478 | Torsin A interacting protein 1 |
| 268 | I3L0M9 | 6005890 | Transcription elongation factor B polypeptide 2  isoform a |
| 269 | P29084 | 4504195 | Transcription initiation factor IIE subunit beta |
| 270 | F5GX39 | 348554277 | Transmembrane emp24 domain-containing protein  2-like |
| 271 | Q69YZ2 | 51242137 | Transmembrane protein 200B |
| 272 | Q92973 | 133925811 | Transportin-1 isoform 1 |
| 273 | E9PHK9 | 207113160 | Treacle protein isoform d |
| 274 | C9J5S7 | 50301248 | TRIM5/cyclophilin A fusion protein |
| 275 | Q53HE2 | 62896835 | Triosephosphate isomerase 1 variant |
| 276 | D3DQU2 | 119589091 | Tripeptidyl peptidase I, Isoform CRA_a |
| 277 | D6RAS3 | 39995082 | TRNA (cytosine(34)-C(5))-methyltransferase isoform 1 |
| 278 | Q9UI30 | 7705477 | TRNA methyltransferase 112 homolog |
| 279 | K7ENT6 | 223555975 | Tropomyosin alpha-4 chain isoform 1 |
| 280 | Q71U36 | 344266823 | Tubulin alpha-1A chain-like |
| 281 | B7ZAF0 | 57209813 | Tubulin, Beta |
| 282 | Q9BV28 | 30582781 | Tubulin, Beta, 4 |
| 283 | B4DR68 | 6525069 | Tumor necrosis factor type 1 receptor associated  protein |
| 284 | P54577 | 114555398 | Tyrosyl-tRNA synthetase, Cytoplasmic isoform 10 |
| 285 | F1T0A5 | 221136939 | U4/U6 small nuclear ribonucleoprotein Prp31 |
| 286 | K7EP67 | 224086292 | U5 snRNP-specific protein, 116 kD |
| 287 | O15116 | 7657313 | U6 snRNA-associated Sm-like protein LSm1 |
| 288 | P54578 | 4827050 | Ubiquitin carboxyl-terminal hydrolase 14 isoform a |
| 289 | B3KSS1 | 150417996 | Ubiquitin-like modifier-activating enzyme 6 |
| 290 | P05161 | 4826774 | Ubiquitin-like protein ISG15 precursor |
| 291 | E9PJ81 | 30923268 | UBX domain-containing protein 1 |
| 292 | E9PBD2 | 291385671 | UDP-glucose dehydrogenase |
| 293 | B4DNS4 | 332256869 | Vesicle-associated membrane protein-associated  protein B/C-like isoform 2 |
| 294 | C9J8H1 | 87159816 | V-type proton ATPase subunit E 1 isoform b |
| 295 | Q9Y2W2 | 7706501 | WW domain-binding protein 11 |
| 296 | O75312 | 4508021 | Zinc finger protein ZPR1 |
| 297 | C9JEV0 | 4502337 | Zinc-alpha-2-glycoprotein precursor |
